# Observation of parity-time symmetry breaking transitions in a dissipative Floquet system of ultracold atoms

**DOI:** 10.1038/s41467-019-08596-1

**Published:** 2019-02-20

**Authors:** Jiaming Li, Andrew K. Harter, Ji Liu, Leonardo de Melo, Yogesh N. Joglekar, Le Luo

**Affiliations:** 1 School of Physics and Astronomy, Sun Yat-sen University, 519082 Zhuhai, China; 2 Department of Physics, Indiana University Purdue University Indianapolis (IUPUI), Indianapolis, IN 46202 USA

## Abstract

Open physical systems with balanced loss and gain, described by non-Hermitian parity-time $\left( {{\cal P}{\cal T}} \right)$ reflection symmetric Hamiltonians, exhibit a transition which could engender modes that exponentially decay or grow with time, and thus spontaneously breaks the ${\cal P}{\cal T}$-symmetry. Such ${\cal P}{\cal T}$-symmetry-breaking transitions have attracted many interests because of their extraordinary behaviors and functionalities absent in closed systems. Here we report on the observation of ${\cal P}{\cal T}$-symmetry-breaking transitions by engineering time-periodic dissipation and coupling, which are realized through state-dependent atom loss in an optical dipole trap of ultracold ^6^Li atoms. Comparing with a single transition appearing for static dissipation, the time-periodic counterpart undergoes ${\cal P}{\cal T}$-symmetry breaking and restoring transitions at vanishingly small dissipation strength in both single and multiphoton transition domains, revealing rich phase structures associated to a Floquet open system. The results enable ultracold atoms to be a versatile tool for studying ${\cal P}{\cal T}$-symmetric quantum systems.

## Abstract (web summary)

Ultracold atoms provide controllable platforms to study many quantum mechanical phenomena. Here the authors use noninteracting fermions of ultracold Li atoms with tunable time‐periodic dissipation or coupling to demonstrate the breaking and restoration of parity‐time symmetry.

## Introduction

A non-Hermitian parity-time reflection symmetric (${\cal P}{\cal T}$-symmetric) Hamiltonian, that is invariant under combined parity $\left( {\cal P} \right)$ and time-reversal $\left( {\cal T} \right)$ operations, has been considered as a natural extension of the conventional Hermitian quantum theory to describe an open quantum system with balanced loss and gain^1–3^. ${\cal P}{\cal T}$-symmetric Hamiltonians exhibit many interesting behaviors^4–9^, in which a key property is ${\cal P}{\cal T}$-symmetry-breaking transitions that occur at an exceptional point^10,11^ – a point in the parameter space where two resonant modes of the Hamiltonian become degenerate. A number of seminal studies^1,12^ have shown that the eigenvalues of the Hamiltonian are real in one side of the transition, allowing the ${\cal P}{\cal T}$-symmetric (PTS) phase, while complex eigenvalues appear in the other side with the ${\cal P}{\cal T}$-symmetric broken (PTSB) phase. In recent years, ${\cal P}{\cal T}$-symmetric Hamiltonians have been realized in balanced gain and loss systems with various setups, such as mechanical oscillators^13^, optical waveguides^14,15^, optical resonators^16^, microwave cavities^17^, lasers^18^, and optomechanical systems^19^, or in a state-dependent pure lossy system in which the lossy Hamiltonian *H*′ could be mapped to a ${\cal P}{\cal T}$-symmetric Hamiltonian *H*_PT_ for passive ${\cal P}{\cal T}$-symmetry breaking^20–22^.

${\cal P}{\cal T}$-transitions can be induced either by increasing the strength of dissipation or by tuning the periodicity of the dissipation, known as static or Floquet method respectively. By driving the system passing the exceptional point, it is predicted that ${\cal P}{\cal T}$-transitions can reduce the overall dissipation of the system^20,23,24^ and allow topological structures around the exceptional point^19,25^. Floquet method is particular interesting because time-periodic modulation can break the continuous time translation symmetry, providing an enriched phase diagram with many fascinating features^20^.

Here we present an experimental study of ${\cal P}{\cal T}$-symmetry-breaking transitions induced by time-periodic dissipation or coupling in a two-spin system of ultracold atoms. Our experimental results verify that ${\cal P}{\cal T}$-symmetry breaking and restoring transitions can occur by tuning either dissipative or coupling frequency even at vanishingly small dissipation strength. We further map the Floquet ${\cal P}{\cal T}$-phase diagrams by tracing the atom loss of each spin state, and observe the multiphoton resonances and the power broadening associated to the PTSB phase.

## Results

### ${\cal P}{\cal T}$ transition with static dissipation

**Fig. 1:**
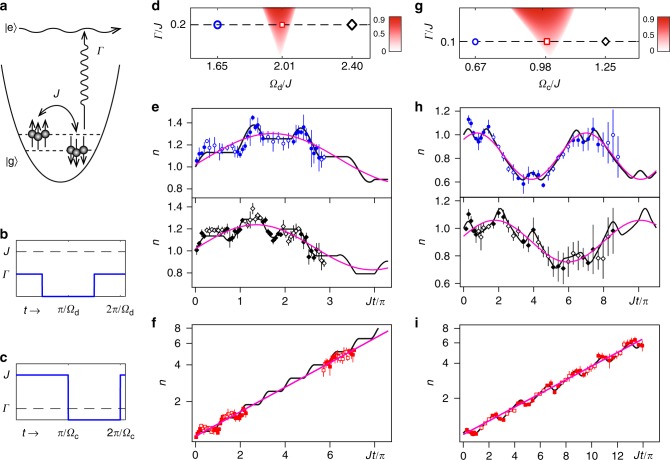
The parity-time transitions induced by time-periodic modulations. **a** Experimental setup. An RF field is used to couple the two-spin states. A resonant optical beam is used to generate spin-dependent dissipation (atom loss) in the |↓〉 state. **b** Γ(*t*) for time-periodic dissipation. **c** *J*(*t*) for time-periodic coupling. **d**–**f** Time-periodic dissipation: **d** The phase diagram near the primary resonance. The red color region represents the ${\cal P}{\cal T}$-symmetric broken (PTSB) phase with (|*μ*_+_| − |*μ*_−_|)/(|*μ*_+_| + |*μ*_−_|) as the value of the color density, as the following figures. **e** *n*(*t*) of the ${\cal P}{\cal T}$-symmetric (PTS) phase at Ω_d_/*J* = 1.65 (blue circles) and Ω_d_/*J* = 2.40 (black diamonds). **f** *n*(*t*) of the PTSB phase at Ω_d_/*J* = 2.01 (red boxes). **g**–**i** Time-periodic coupling: **g** The phase diagram near the primary resonance. **h** *n*(*t*) of the PTS phase at Ω_d_/*J* = 0.67 (blue circles) and Ω_d_/*J* = 1.25 (black diamonds). **i** *n*(*t*) of the PTSB phase at Ω_d_/*J* = 0.99 (red boxes). In **e**, **f**, **h**, **i**, the data with solid shapes corresponds with the dissipation (coupling) on, and the data with empty shapes are with the dissipation (coupling) off. In all figures, the black curves are the numerical simulation without free parameter, and the pink curves are the sinusoidal (for PTS) or exponential (for PTSB) fitting. *J* = *π* × 2.15 kHz for all data presented in this paper if not mentioned. The error bars are the standard deviation of the measurements

We prepare a noninteracting Fermi gas of ^6^Li atoms at the two lowest ^2^S_1/2_ hyperfine levels^26,27^, labeled as |↑〉 and |↓〉. These two-spin states are coupled by a radio-frequency (RF) field with a coupling strength of *J*. A resonant optical beam is used to excite the atoms from |↓〉 to the 2P excited state $\left| e \right\rangle$ and generates the atom loss in |↓〉 with a rate of Γ (Fig. 1a). The Hamiltonian for this dissipative two-spin system is given by

1 $$H(t) = J\sigma _{\mathrm{x}} - i\Gamma (t)\left| \downarrow \right\rangle \left\langle \downarrow \right| = - i{\mathrm{\Gamma }}(t){\mathrm{/}}2{\bf{I}} + H_{{\mathrm{PT}}}(t)$$

where *H*_PT_(*t*) = *Jσ*_x_ + *i*Γ(*t*)*σ*_z_/2 is a ${\cal P}{\cal T}$-symmetric Hamiltonian, and **I** is the unit matrix. The system is prepared with all atoms in |↑〉, and evolves for a time of *t*. Then the in-trap atom numbers, $n_ \uparrow ^\prime (t)$ and $n_ \downarrow ^\prime (t)$, are measured by the double-shot absorption imaging of the two-spin states, giving the total atom number $n{\prime}(t) = n_ \uparrow ^\prime + n_ \downarrow ^\prime$. We map *n*′(*t*) to a scaled, normalized atom number *n*(*t*) associated to *H*_PT_, and then use *n*(*t*) to characterize the ${\cal P}{\cal T}$-transitions [See Methods].

For static dissipation, Γ(*t*) is a constant value of Γ_0_. When Γ_0_/*J* < 2, the eigenvalues of *H*_PT_ are real values of $\pm \sqrt {J^2 - {\mathrm{\Gamma }}_0^2{\mathrm{/}}4}$, and *n*(*t*) oscillates at frequency $\pi {\mathrm{/}}\sqrt {J^2 - \Gamma _0^2{\mathrm{/}}4}$. The ${\cal P}{\cal T}$ transition occurs at Γ_0_/*J* = 2 where the oscillation period diverges. When Γ_0_/*J* > 2, the eigenvalues of *H*_PT_ become complex numbers, and one of the eigenmode exponentially grows [See Supplementary Note 1]. These predictions are verified in our experiments [See Supplementary Fig. 1], and the measured exceptional point agrees with the theoretical model very well [See Supplementary Fig. 2].

The static dissipation experiment is related to the previous quantum zeno effect (QZE) experiments of ultracold atoms with strong-loss induced measurement^28–31^. In those experiments, QZE refers to the reduction of the rate of transferring from one state to a second state by the projection measurement of the second state. Due to a strong-loss induced irreversible measurement, the reverse-transfer probability from the second state to the first one is treated as zero as well as the occupation of the second state. However, in our dissipation experiment, the transfer probability from the second to the first level is nonzero, and the PTSB phase refers to the slow-down of the decay of the total atom number. Thus, the results cannot be explained purely in terms of QZE, except for the limit of an extremely strong dissipation case, in which the strong atom loss can be treated as an irreversible projection measurement of the second level.

### Observation of the ${\cal P}{\cal T}$-transitions with time-periodic driving

Floquet method enriches the phase diagram of a ${\cal P}{\cal T}$-symmetric system by periodically modulating Hamiltonian *H*(*t*) = *H*(*t* + *T*). Previously, the extraordinary structure of the phase diagram has been theoretically predicted^20,23^, but has never been verified experimentally due to the difficulty of precisely controlling the time-dependent dissipation. In our experiment, the optical and RF field provide versatile tools to manipulate the atom loss and coupling of spin levels, so that two types of Floquet Hamiltonians could be implemented: spin-dependent time-periodic dissipation and time-periodic coupling between two spins.

We first study time-periodic dissipation, in which a square-wave resonant beam is applied to generate time-dependent dissipation of the atoms in an optical trap. The coupling strength *J* is fixed and the dissipation strength is modulated between Γ and 0 with a frequency of Ω_d_ (Fig. 1b). In contrast with the static dissipation, ${\cal P}{\cal T}$-transitions under time-periodic dissipation depends on the modulation frequency and can occur at vanishingly small dissipation strength with infinite numbers of the resonance peaks [See the Supplementary Note 2]. The primary resonance peak of the ${\cal P}{\cal T}$-transition appears Ω_d_/*J* = 2, where the transition behavior of *n*(*t*) in the weak dissipation limit ${\mathrm{\Gamma /}}J = 0.2 \ll 2$ is shown in Fig. 1d–f. When Ω_d_/*J* is tuned to the PTSB phase, *n*(*t*) increases exponentially, in contrast with the PTS phase where *n*(*t*) exhibits bounded oscillation *n*(*t*) ∝ sin[(Ω_d_ − 2*J*)*t*].

In the above cases, the PTSB phases have been observed even when the eigenvalues of *H*(*t*) are real all the time. Such ${\cal P}{\cal T}$-symmetry breaking can be determined by the non-unitary time-evolution operator *G*_PT_ [See Methods], which has two eigenvalues $\mu _ \pm \propto e^{ - i\epsilon _ \pm t}$. $\epsilon _ \pm$ is the quasienergies of the effective Floquet Hamiltonian [See Supplementary Note 3]. If the magnitude of *μ*_±_ are equal, $e^{i\epsilon _ \pm T}$ is a pure phase factor and $\epsilon _ \pm$ must be the real numbers, which indicate a PTS phase. On the contrary, the unequal magnitude of *μ*_±_ denote the complex values of $\epsilon _ \pm$ representing a PTSB phase.

For time-periodic coupling in the weak dissipation limit, Γ/*J* is constant and *J*(*t*) is modulated at the frequency Ω_c_ (Fig. 1c) and the the primary resonance peak of the PTSB phase is at Ω_c_/*J* = 1 (Fig. 1g). When Ω_c_/*J* is tuned to the primary resonance region, *n*(*t*) shows the similar behavior as time-periodic dissipation, where the exponential increase of *n*(*t*) appears (Fig. 1i), while the PTS phase exhibits the bound oscillation which could be parameterized by *n*(*t*) ∝ sin[(Ω_c_ − *J*)*t*] in the weak dissipation limit (Fig. 1h). The measurements of that primary resonance of the PTSB phase verify that ${\cal P}{\cal T}$-symmetry transitions can happen with an arbitrary small dissipation under time-periodic driving. Furthermore, there exist infinite numbers of transitions induced by multiphoton resonances which are investigated as follows.

### Multiphoton resonances with time-periodic dissipation

**Fig. 2:**
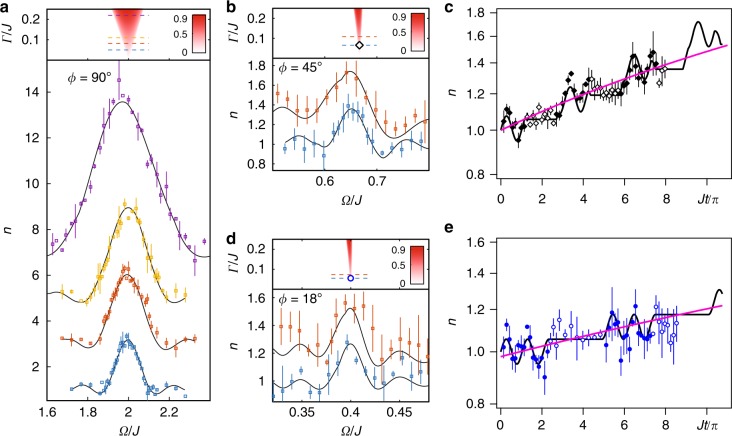
Detecting the resonances with time-periodic dissipation. **a** *n*(*t*_f_, Ω) around the one-photon resonance Ω_n_ = 2*J*. Purple for Γ/*J* = 0.22, *t*_f_ = 1.98 ms; Yellow for Γ/*J* = 0.11, *t*_f_ = 3.31 ms; Red for Γ/*J* = 0.082, *t*_*f*_ = 3.97 ms; and Blue for Γ/*J* = 0.05, *t*_f_ = 5.29 ms. **b** *n*(*t*_f_, Ω) around the three-photon resonance Ω_n_ = 2*J*/3. Red for Γ/*J* = 0.11, *t*_f_ = 2.80 ms; Blue for Γ/*J* = 0.065, *t*_f_ = 3.79 ms. **c** *n*(*t*) at Ω_n_ = 2*J*/3 with Γ/*J* = 0.065 located in (**b**) by the diamond shape. **d** *n*(*t*_f_, Ω) around the five-photon resonance Ω_n_ = 2*J*/5. Red for Γ/*J* = 0.07, *t*_f_ = 4.64 ms; Blue for Γ/*J* = 0.055, *t*_f_ = 4.74 ms. **e** *n*(*t*) at Ω_n_ = 2*J*/5 with Γ/*J* = 0.055 located in (**d**) by the circle shape. The initial phase *ϕ* of the square-wave modulation is chosen to anti-synchronize to the RF field with *ϕ* = *π*/2, *π*/4, *π*/10 for one-, three-, and five-photon resonance respectively. In **a**, **b**, and **d**, all the data and simulation curves have a base line of *n* = 1, but are vertically shifted for the presentation purpose. The side peaks are due to the finite probe time. In **c**, **e**, the data with solid shapes corresponds with the dissipation on, the data with empty shapes are with the dissipation off, and the pink curves are the exponential fitting. For all figures, the solid curves are numerical simulations without free parameter. The error bars represents the standard deviation of the measurements

For ${\cal P}{\cal T}$-transitions with time-periodic dissipation, there exist infinite numbers of the PTSB phases induced by multiphoton process in a non-Hermitian Rabi model^20^. Their widths have been predicted to decrease with the index number of the the resonances. For a square-wave modulation, the widths of the PTSB phases in the weak dissipation limit are

2 $$\delta {\mathrm{\Omega }}_{\mathrm{d}}({\mathrm{\Gamma }},{\mathrm{\Omega }}_{\mathrm{n}}) = \frac{{\mathrm{\Gamma }}}{\pi }\left( {\frac{{{\mathrm{\Omega }}_{\mathrm{n}}}}{{2J}}} \right)^2,$$

where Ω_n_ = 2*J*/*n* is the resonance peak under zero dissipation with *n* as the odd number 1, 3, 5 …. Γ is the magnitude of the square-wave dissipation [See Supplementary Note 2]. Figure 2 show the broadening of the PTSB phases for one- (primary), three-, and five-photon resonances. To measure the width of the resonances, the residual atom number *n*(*t*_f_, Ω) is probed at a fixed time point *t*_f_ for various modulation frequencies Ω [See Supplementary Note 4]. It is noted that, for the purpose of mapping the phase diagram, it is ideal to choose *t*_f_ as large as possible so that *n*(*t*_f_) can reflect the long-term dynamics. However, because we map a pure lossy system to a ${\cal P}{\cal T}$-symmetric Hamiltonian, *t*_f_ must be remained in a finite range for the reasonable signal-to-noise ratio of the unscaled atom number *n*′(*t*) [See Supplementary Note 5]. In our experiment, we choose *t*_f_ to be larger than several oscillation periods so that *n*(*t*_f_, Ω) can present the trend of increasing in the PTSB phase. As shown in Fig. 2a, the half width at half maximum (HWHM) of the primary resonance is proportional to the strength of time-periodic dissipation. Such behavior is the non-Hermitian analog of the resonance broadening induced by the Bloch–Siegert shifts of a strong driving Hermitian system. The width of the residual atom number also depends on the probe time and gets narrower for the longer probing times, which approaches the width of the PTSB phase predicted by theoretical calculations. For the finite probe time, the width is a qualitatively measure of the dependence of the PTSB regime on the dissipation strength [See Supplementary Note 4].

Comparing with the Hermitian system where the multiphoton resonance is difficult to be observed at the weak driving limit, in a non-Hermitian system, the time-periodic dissipation significantly broadens the width of the PTSB phase so that the multiphoton resonance could be observed clearly with the weak dissipation. The widths of the three-(Fig. 2b), five- (Fig. 2d) photon resonances, agree with the theoretical phase diagram very well. At the exact resonant frequencies, the exponentially increase of *n*(*t*) with a very small dissipation strength are recorded in Fig. 2c (three-photon) and Fig. 2e (five-photon) manifesting the PTSB phase.

### Multiphoton resonances with time-periodic coupling

**Fig. 3:**
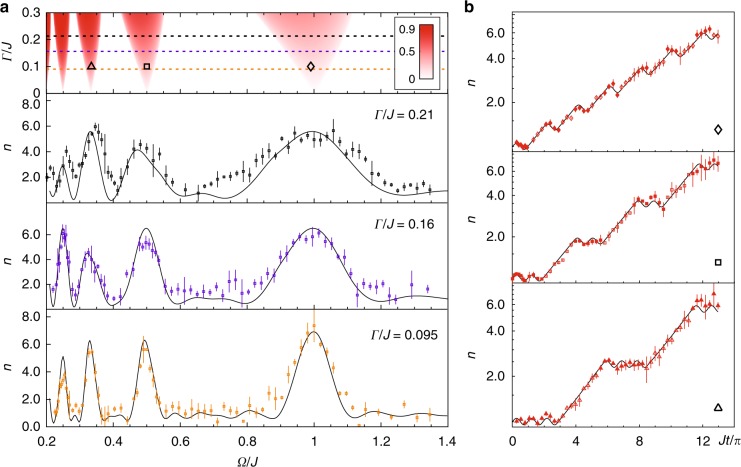
Detecting multiphoton resonances with time-periodic coupling. **a** The top frame is the phase diagram. *n*(*t*_f_, Ω) are shown below. From the second top frame to the bottom one, Γ/*J* = 0.21, 0.16, 0.095, *t*_f_ = 2.70, 3.70,6.48 ms respectively. The side peaks are mainly due to the finite probe time. **b** *n*(*t*) with Γ/*J* = 0.095 at the resonant peak Ω_n_ = *J*, *J*/2, *J*/3 from the top to bottom frame. The data with solid shapes corresponds with the dissipation on, the data with empty shapes are with the dissipation off. For all figures, solid curves are the numerical simulation without free parameters. The initial phases of the square-wave modulation are set to zero. The error bars represent the standard deviation of the measurements

The phase diagram of time-periodic coupling is studied by modulating the coupling between the two-spin states. The resonance widths of the PTSB phases are given by

3 $$\delta {\mathrm{\Omega }}_{\mathrm{c}}({\mathrm{\Gamma }},{\mathrm{\Omega }}_{\mathrm{n}}) = {\mathrm{\Gamma }}\frac{{{\mathrm{\Omega }}_{\mathrm{n}}}}{{2J}},$$

where Ω_n_ = 2*J*/*n* is the resonant peak with the even integer *n* = 2, 4, 6 .... Eq. (3) indicates that the PTSB phases of the time-periodic coupling have the wider width than that of time-periodic dissipation, which scales with the multiphoton index number *n* as 1/*n* instead of 1/*n*^2^ for time-periodic dissipation [See Supplementary Note 2]. The first four multiphoton resonances are shown in Fig. 3a, where the widths of the PTSB phases increase with dissipation. The increasing of *n*(*t*) at the resonance frequencies are fitted by the exponential curves in Fig. 3b to verify the PTSB phase.

It is interesting to find that, the resonant peaks Ω_n_ = 2*J*/*n* of the time-periodic dissipation have odd integers *n* = 1, 3, 5 ..., but the time-periodic coupling ones have even integers *n* = 2, 4, 6 .... The odd or even rule can be explained by a simple picture. For example, in the time-periodic coupling case of the weak loss limit, all atoms are initially in the lossless (up) state and the coupling is turned on for *nπ*/(2*J*). If n is even, then the coupling is on exactly for the time of multiple 2*π* Rabi pulses such that all atoms are back to the up state, and will remain in this state for the next half cycle of coupling-off. During this half cycle, no atoms are lost so that the scaled total atom number increase because the scaling assumes equal loss in both spin states. Overall, the system spends more time in the lossless (up) state when *n* is even numbers. This is not the case for n to be odd, where on average the system spends the same amount of time in both up and down states because the coupling is turned on for the odd numbers of *π* pulse.

The similar picture is also applied to the time-periodic dissipation case, where the atom loss is turned on for a time duration of *nπ*/(2*J*) with *n* being odd numbers. This amount of time is odd numbers of *π* Rabi pulse. With the proper choice of phase, the atom loss can only present when the majority of the atoms are in the lossless (loss) state so that the scaled total atom number increase (decrease) exponentially. Either increasing or decreasing depends on the phase of the dissipation, which corresponds the two eigenstates in the PTSB phase. In the experiments, we usually optimize the phase of the time-periodic modulation to obtain the strongest signal, resulting from ensuring the largest overlap between the initial state and the slowly decaying eigenmode of the corresponding Floquet Hamiltonian. For more general initial states, such as a balanced mixture of up and down state, as long as there is a nonzero overlap between the initial state and the slow mode, the ${\cal P}{\cal T}$-symmetry-breaking signatures of the slow-decaying will be visible in the long-time limit.

## Discussion

The laser-cooled ultracold atoms provide a clean and well controllable platform for studying ${\cal P}{\cal T}$-symmetric Hamiltonians. Previously, phase transitions observed in cold atom systems are usually driven by tunable interparticle interactions and, in principle, occur only in the thermodynamic limit. However, ${\cal P}{\cal T}$-symmetric breaking transitions, in contrast, can occur in a single two-level system with localized loss. The fate of the former transitions in the presence of such a loss has not been fully understood, as is the fate of latter transition in the presence of interparticle interactions. Investigating the interplay between these two classes of transitions will require quantum simulators with tunable interparticle interactions and engineered state-dependent dissipation, both of which can be realized with certain species of ultracold atoms, such as fermionic ^6^Li atoms used in this experiment. As a starting point of this route, we apply state-dependent dissipation to ultracold ^6^Li atoms to study ${\cal P}{\cal T}$-transitions in a two-level system. With the advantages of modulating the resonant optical and RF field as the versatile tools for time-periodic driving, we could manipulate the atom loss as well as the coupling of spin levels, and experimentally map both time-periodic dissipation and coupling. The phase diagram of the static dissipation and time-periodic dissipation (coupling) are explored by tracing the time-evolution of the atoms. While the single exceptional point under static dissipation is determined as usual, our results verify remarkably rich phase diagrams with multiple Floquet ${\cal P}{\cal T}$-transitions associated to time-periodic driving. It is shown that the PTSB phases can be induced by judiciously selected temporal profiles of state-dependent dissipation or coupling with vanishingly small strength of the dissipation. The multiphoton resonant structure of ${\cal P}{\cal T}$-transitions are demonstrated. Such Floquet method thus provide an experimental platform to study time-dependent ${\cal P}{\cal T}$-symmetric Hamiltonians.

Our system has potential to be extended to more complex situations: one is to study the topological phenomena associated to non-Hermitian Hamiltonian and the other is to explore an interacting system with a vanishingly small, time-modulated dissipation. For the formal one, if we use the unresonant RF pulses to couple the spin levels, a detuning term will appear in the diagonal part of the Hamiltonian, and we can adiabatically encircle the exceptional points by changing the detuning and dissipation simultaneously to observe the topological phenomena associated to the non-Hermitian systems^19,25,32–38^. For the latter one, the interplay between the ${\cal P}{\cal T}$-transition and the BEC-BCS (Bose–Einstein condensate to Bardeen–Cooper–Schrieffer pairing) crossover can be investigated by sweeping the ultracold Fermi gas from the noninteracting limit (presented here) to the unitary, strongly-interacting limit^39^. This approach, where a single-particle, state-dependent loss is used in conjunction with strong interparticle interactions, provides exciting opportunities to explore physical phenomena in open many-body quantum systems.

## Methods

### Experimental system

We prepare a dissipative two-level system with a noninteracting Fermi gas. ^6^Li atoms are prepared in the two lowest hyperfine states, |↑〉 ≡ |F = 1/2, *m*_F_ = 1/2〉 and |↓〉 ≡ |F = 1/2, *m*_F_ = −1/2〉, in a magneto-optical trap. The precooled atoms are then transferred into a crossed-beam optical dipole trap made by a 100 Watt fiber laser. The bias magnetic field is swept to 330 G to implement an evaporative cooling^26^. The trap potential is lowered to generate a final trap depth of 2.2 *μ*K. In order to null the interaction between the two hyperfine states, the magnetic field is fast swept to 527.3 G, where the s-wave scattering length of the |↑〉 and |↓〉 states is zero^27^. The lifetime of the noninteracting Fermi gas is about 20 s, which is three orders of magnitude longer than our typical experimental time. So when the dissipative optical field is absent, this noninteracting Fermi gas can be treated as a closed, two-level quantum system. To prepare a single component Fermi gas in the |↑〉 state as the initial state, we apply a 5 ms optical pulse with −2*π* × 30 MHz detuning from the |↓〉 → ^2^P_3/2_ transition to blow away atoms in the |↓〉 state. We typically have about *N* = 2.0 × 10^5^ atoms in a pure |↑〉 state at temperature *T* ≈ 0.8 *μ*K and *T*/*T*_F_ ≈ 0.5 with *T*_F_ is the Fermi temperature.

**Fig. 4:**
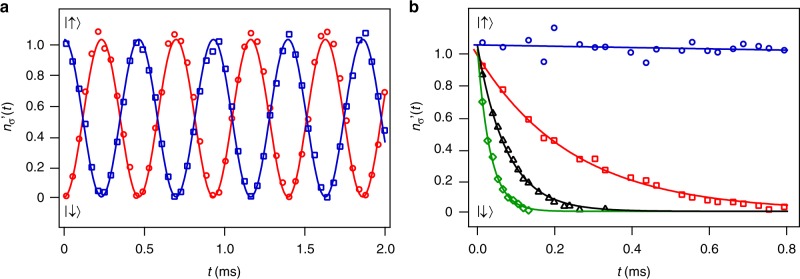
Characterization of a dissipative Rabi system. Symbols are experimental data. Solid lines are theoretical fits. **a** In the presence of the RF field but without the dissipative optical field, atom numbers $n_\sigma ^\prime (t)$ show Rabi oscillations with a Rabi frequency of 2*J* = (2*π*) × 2.15 kHz. **b** In the presence of an optical field resonant with |↓〉 but without the RF field, $n_ \downarrow ^\prime (t)$ shows an exponential decay: Γ = 0.30*J* (red squares), Γ = 0.98*J* (black triangles), Γ = 2.35*J* (green diamonds). $n_ \uparrow ^\prime (t)$ remains almost constant during the experimental time. Blue circles show $n_ \uparrow ^\prime (t)$ when Γ = 0.30*J*

To generate Rabi oscillation between the two-spin states, we couple them via an RF field with frequency *ω* and coupling strength *J*. An optical beam resonant with the |↓〉 → ^2^P_3/2_ transition is used to create the number dissipation (atom loss) in the |↓〉 state. The resonant-photon recoil energy of 3.5 μK is ~50% larger than the trap depth, so the atom that absorb a photon will leave the trap quickly, resulting a state-dependent atom loss. The RF coupling strength *J* is measured in the absence of the dissipative optical field, while the atom-number loss rate 2Γ is measured in the absence of the RF coupling. Figure 4a shows the Rabi oscillation with Rabi frequency 2*J*. Figure 4b shows the atom numbers $n_ \downarrow ^\prime (t) = n_ \downarrow ^\prime (0){\mathrm{exp}}( - 2{\mathrm{\Gamma }}t)$ with a constant dissipative optical field that only couples the |↓〉 state to the continuum. These measurements are used to calibrate the values of *J* and Γ for the dissipative two-state Rabi system.

### Theoretical model

The dissipative two-state system is described by a non-Hermitian Hamiltonian (*ħ* = 1)

4 $$H = + \frac{{\omega _0}}{2}\sigma _{\mathrm{z}} - i\frac{{{\mathrm{\Gamma }}(t)}}{2}(1 - \sigma _{\mathrm{z}}) + 2J\,{\mathrm{cos}}(\omega t)\sigma _{\mathrm{x}},$$

where *ω*_0_ = 2*π* × 75.6 MHz is the hyperfine splitting at 527.3 G. When the RF driving is close to the resonance, that is *ω* ≈ *ω*_0_, with the rotating wave approximation in the interacting picture, *H*(*t*) = −*i*Γ(*t*)/2 + *H*_PT_(*t*), where the non-Hermitian, ${\cal P}{\cal T}$-symmetric Hamiltonian is given by (*ħ* = 1) $H_{{\mathrm{PT}}} = J\sigma _{\mathrm{x}} + i{\mathrm{\Gamma }}(t)\sigma _{\mathrm{z}}{\mathrm{/}}2 = {\cal P}{\cal T}H_{{\mathrm{PT}}}{\cal P}{\cal T}$, where ${\cal P} = \sigma _{\mathrm{x}}$ and ${\cal T} = \ast$ denotes complex conjugation operation. Starting with an initial state |*ψ*(0)〉, the decaying atom numbers for the two states are given by $n_\sigma ^\prime (t) \equiv \left| {\left\langle \sigma \right|G\prime (t)\left| {\psi (0)} \right\rangle } \right|^2$ where

5 $$G\prime (t) = T\,{\mathrm{exp}}\left( { - i{\int}_0^t \,H\prime (t\prime )dt\prime } \right),$$

is the non-unitary time-evolution operator obtained via the time-ordered product. It is also useful to define scaled atom number *n*_*σ*_(*t*) = |〈*σ*|*G*_PT_(*t*)|*ψ*(0)〉|^2^ where *G*_PT_(*t*) is the corresponding time-evolution operator for *H*_PT_(*t*). It follows that $n_\sigma (t) = n_\sigma ^\prime (t) \times {\mathrm{exp}}\left( {{\int}_0^t {\kern 1pt} {\mathrm{\Gamma }}(t\prime )dt\prime {\mathrm{/}}2} \right)$. In a ${\cal P}{\cal T}$-symmetric system, the ${\cal P}{\cal T}$-symmetric phase is signaled by non-decaying, oscillatory *n*_*σ*_(*t*) and the ${\cal P}{\cal T}$-broken phase is signaled by an exponentially increasing *n*_*σ*_(*t*).

## Supplementary information

Supplementary Information

## Data Availability

The data that support the findings of this study are available from the corresponding author upon reasonable request.
