# Supplementary material for: Observation of parity-time symmetry breaking transitions in a dissipative Floquet system of ultracold atoms
Source: Nat Commun. 2019 Feb 20;10:855. doi: 10.1038/s41467-019-08596-1 (PMC6382795; doi:10.1038/s41467-019-08596-1)
Supplement: Supplementary file 1 — Supplementary Information [file 41467_2019_8596_MOESM1_ESM.pdf]

Observation of parity-time symmetry breaking transitions in a dissipative Floquet system of ultracold atoms

Jiaming Li et al.[1,2,*]

[1]*School of Physics and Astronomy, Sun Yat-sen University, Zhuhai 519082, China*

[2]*Department of Physics, Indiana University Purdue University Indianapolis (IUPUI), Indianapolis, Indiana 46202, USA*

Supplementary Note 1: Passive $\mathcal{PT}$-symmetry breaking transition with static dissipation

When the resonant optical field is constant in time, that is $\Gamma(t) = \Gamma_0$, the eigenvalues of the dissipative Hamiltonian H are given by $-i\Gamma_0/2 \pm \lambda = -i\Gamma_0/2 \pm \sqrt{J^2 - \Gamma_0^2/4}$. Starting with the initial state $|\psi(0)\rangle = |\uparrow\rangle$, the atom numbers for the two levels are given by

$$\begin{aligned} n'_\uparrow(t) &= e^{-\Gamma_0 t} \left|\cos(\lambda t) + (\Gamma_0/2\lambda)\sin(\lambda t)\right|^2, \\ n'_\downarrow(t) &= e^{-\Gamma_0 t} \left|i(J/\lambda)\sin(\lambda t)\right|^2. \end{aligned} \tag{1}$$

The corresponding scaled atom numbers are given by $n_\sigma(t) = e^{\Gamma_0 t} n'_\sigma(t)$. The passive $\mathcal{PT}$-symmetric breaking transition occurs at $\Gamma_0 = 2J$. When $\Gamma_0 < 2J$, λ is real and $n_\sigma(t)$ oscillates with a period of π/λ which increases as Γ_0 increases. When $\Gamma_0 > 2J$, λ becomes purely imaginary, leading to the two eigenmodes with different decay rates, one of which decreases as $(2J)^2/\Gamma_0$. So when Γ_0 increase, the total unscaled atom number $n'(t)$ decays slower. Correspondingly, the scaled atom number $n(t) = n_\uparrow(t) + n_\downarrow(t)$ increases exponentially with time.

Supplementary Figure 1a shows the total atom number $n'(t)$ for various static dissipation. For smaller dissipation in the PTS phase, the atom number $n'(t)$ decays faster as Γ_0 is increased (blue circles and diamonds); this trend is reversed in the PTSB phase, that is $\Gamma_0 > 2J$ (red squares). Thus, the passive $\mathcal{PT}$-symmetry breaking transition occurs when $n'(t)$ vanishes most rapidly. Supplementary Figure 1b shows the same data, plotted in terms of the scaled atom number $n(t)$. As is expected, $n(t)$ shows oscillatory behavior with increasing amplitude and period in the PTSB phase (blue circles and diamonds). Such behavior gives way to exponential-in-time in the PTSB phase (red squares).

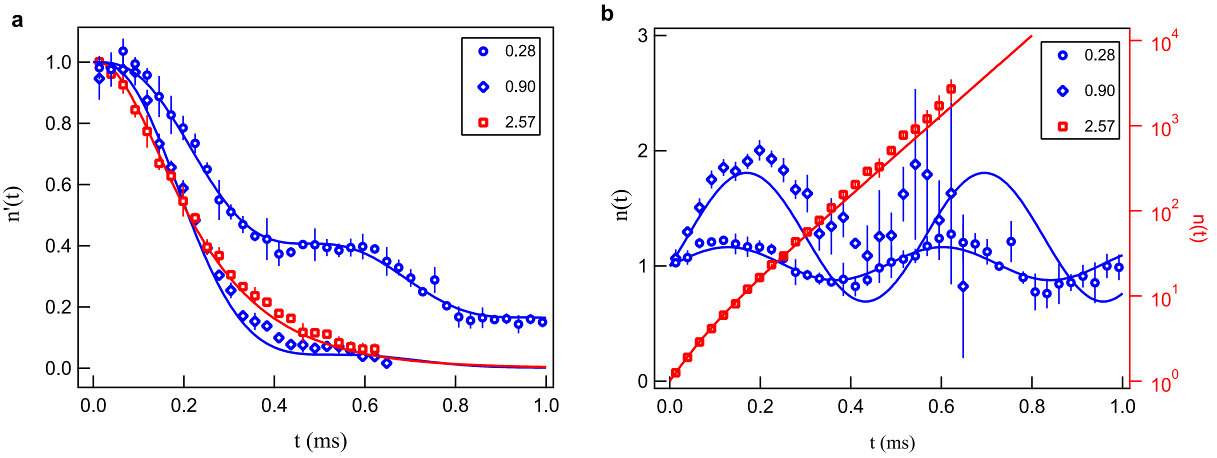

Supplementary Figure 1: Observation of the passive $\mathcal{PT}$ transition with static dissipation. **a** $n'(t)$ shows faster decay as the loss strength is increased from $\Gamma_0 = 0.28J$ (blue circles) to $\Gamma_0 = 0.9J$ (blue diamonds) in the PTS phase. This trend is reversed at large $\Gamma_0 = 2.57J$ (red squares) indicating a PTSB phase. Here $J = \pi \times 2.15$ kHz. **b** $n(t)$, obtained from the same data, shows oscillations with increasing amplitude and period in the PTS phase and exponential rise (right vertical axis) in the PTSB phase. Each data point denotes average over 6 single-shot measurements and the error bars are the standard deviation of the measurements.

To determine the passive $\mathcal{PT}$ phase transition threshold, we track the individual-level atom numbers $n'_\sigma(\Gamma_0)$ and their sum $n'(\Gamma_0)$ at a fixed time $t_m = \pi/2J$ as a function of dissipation strength in Supplementary Figure 2. $n'(\Gamma_0)$ first decreases and then increases, due to the emergence of the long-lived mode in the PTSB phase. We fit these fixed-time data $n'_\sigma(\Gamma_0)$ to supplementary Eq.(1), with a fitting parameter for the $\mathcal{PT}$-threshold, that is $\lambda = \sqrt{\Gamma_{\rm exp}^2 - \Gamma_0^2}/2$, and extract a threshold value $\Gamma_{\rm exp} = (1.92 \pm 0.09)J$, which matches the theoretical value $\Gamma_{\rm exp} = 2.0J$ well shown in the inset in Supplementary Figure 2.

Supplementary Note 2: Phase diagram of Floquet $\mathcal{PT}$-symmetry transition

$\mathcal{PT}$ transition phase diagram with time-periodic dissipation. A perturbative calculation shows that for a sinusoidal modulation of dissipation, the PTSB phase appearers near the modulation frequencies $\Omega \in [\Omega_{\rm n} - \delta\Omega_{\rm n}, \Omega_{\rm n} + \delta\Omega_{\rm n}]$,

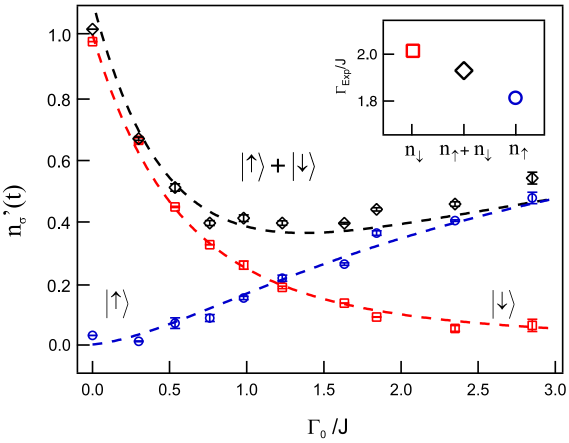

Supplementary Figure 2: Determination of the $\mathcal{PT}$ threshold. Individual level and total atom numbers $n'_\sigma(\Gamma_0)$ are measured at a fixed time $t_\mathrm{m} = \pi/(2J)$ as a function of static dissipation strength. $n'(\Gamma_0)$ shows non-monotonic behavior indicating the passive $\mathcal{PT}$ transition. The symbols are experimental data averaged over 5 single-shot measurements and the dashed lines are the fitting results with Γ_exp as the fitting parameter. The inset shows Γ_exp obtained from the best-fits of the data. In all cases, the initial state of the system is $|\uparrow\rangle$. The error bars are the standard deviation of the measurements.

where

$$\delta\Omega_\mathrm{n} = \frac{(\Gamma_0)^{2n+1}}{2\pi n(2n+1)(4J)^{2n}}. \tag{2}$$

The half-width $\delta\Omega_\mathrm{n}(\Gamma_0)$ decreases as the power-law with the order of the resonances.

It is difficult to implement the sinusoidal modulation precisely in the experiment because the nonlinear relation between the intensity of the resonant beam and the atom loss strength $\Gamma(t)$, so we use a square-wave loss profile with period $2\tau = 2\pi/\Omega$ as,

$$\Gamma(t) = \begin{cases} \Gamma_0 & 0 \le t < \pi/2\Omega, \\ 0 & \pi/2\Omega \le t < 3\pi/2\Omega, \\ \Gamma_0 & 3\pi/2\Omega \le t \le 2\pi/\Omega. \end{cases} \tag{3}$$

The non-unitary time evolution operator

$$G_{PT}(2\pi/\Omega) \equiv e^{-iH(t+\tau,t+2\tau)} e^{-iH(t,t+\tau)} \tag{4}$$

can be analytically calculated for square-wave modulation, and we can use it to determine the phase diagram.

The numerical $\mathcal{PT}$ phase diagram is shown in Supplementary Figure 3 as plotted in the (Γ_0, Ω) plane. In the weak-loss limit, the eigenvalues of $G_{PT}(2\pi/\Omega)$ are given by

$$\mu_\pm(\gamma,\omega) = \cos(2\pi/\omega) \pm i\left[\sin^2(2\pi/\omega) - \gamma^2\sin^2(\pi/\omega)\right]^{1/2} \tag{5}$$

where $\gamma = \Gamma_0/2J \ll 1$ is the dimensionless loss amplitude and $\omega = \Omega/J$ is the dimensionless frequency. The system is in the PTSB phase when the two eigenvalues have different magnitudes, which happens when $\Omega \in [\Omega_\mathrm{n} - \Delta\Omega_\mathrm{n}, \Omega_\mathrm{n} + \Delta\Omega_\mathrm{n}]$ where

$$\Delta\Omega_\mathrm{n}(\Gamma_0) = \frac{\Gamma_0}{\pi}\left(\frac{\Omega_\mathrm{n}}{2J}\right)^2. \tag{6}$$

In comparison with the sinusoidal case [1], the half-width $\Delta\Omega_\mathrm{n}(\Gamma_0)$ of the square-wave modulation remains linearly proportional to the loss amplitude. Supplementary Figure 3b shows the vicinity of first three resonances $\Omega_\mathrm{n} = \{2J/5, 2J/3, 2J\}$ from the numerical simulation as well as the analytical calculation of the phase boundaries (white lines)from supplementary Eq.(6). As n increases, both Ω_n and the half-width of the PTSB phase $\Delta\Omega_\mathrm{n}$ decrease. Note that the linear-phase-boundary is a better approximation for smaller loss amplitude.

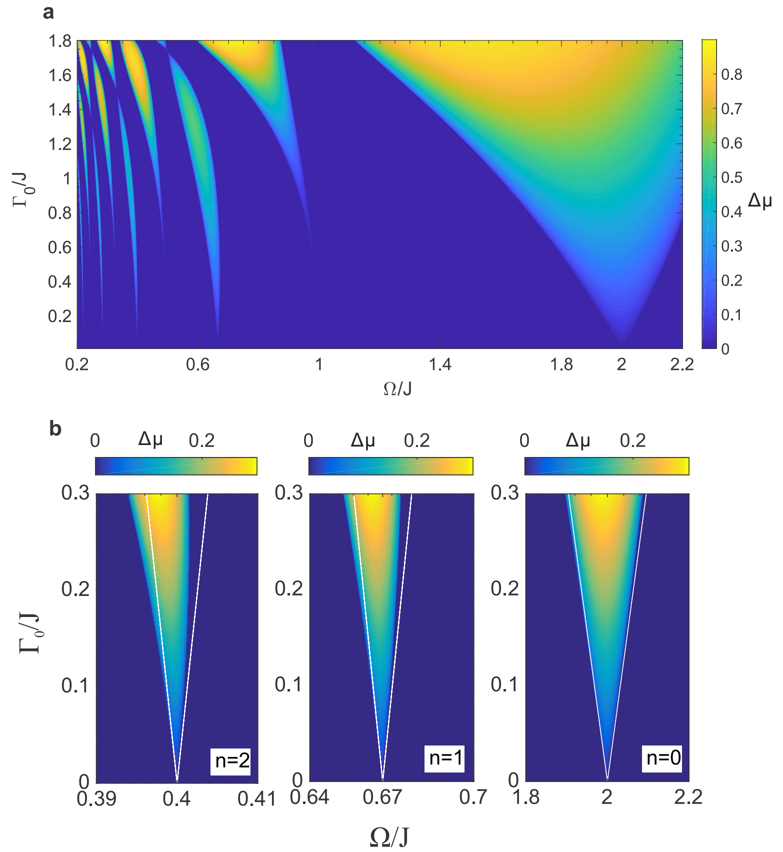

Supplementary Figure 3: $\mathcal{PT}$ phase diagram of the square-wave modulated time-periodic dissipation. The color region represents the PTSB phase with $\Delta\mu = (|\mu_+| - |\mu_-|)/(|\mu_+| + |\mu_-|)$ as the value of the color density. **a** In the range of $\Omega/J \in \{0.2, 2.2\}$ and $\Gamma_0/J \in \{0.01, 1.8\}$. **b** In the vicinity of first three resonances, $\Omega_\text{n} = 2J/(2n+1)$ for $n = \{2, 1, 0\}$. The triangular region of the PTSB phase ($\Delta\mu > 0$) separates the PTS phase ($\Delta\mu = 0$). White lines are the phase boundaries obtained from supplementary Eq.(6) which are good approximation in the limit $\Gamma_0 \ll 2J$.

$\mathcal{PT}$ transition phase diagram with time-periodic coupling. We also apply the square-wave modulation on the coupling strength of two spin states. For time-periodic coupling experiments, we apply constant dissipation Γ_0, but modulate $J(t)$ with a square-wave. Applying the similar method to get the analytical eigenvalues $G_{PT}(2\pi/\Omega)$ in the weak-loss limit, we have

$$\mu_\pm(\gamma,\omega) = \cos\frac{\pi}{\omega}\cosh\frac{\gamma\pi}{\omega} + 2\gamma\sin\frac{\pi}{\omega}\sinh\frac{\gamma\pi}{\omega} \pm [-\sin^2\frac{\pi}{\omega} + (\cos\frac{\pi}{\omega}\sinh\frac{\gamma\pi}{\omega} + 2\gamma\sin\frac{\pi}{\omega}\cosh\frac{\gamma\pi}{\omega})^2]^{1/2} \tag{7}$$

Following supplementary Eq.(7), the system is in the PTSB when the two eigenvalues have different magnitudes, which happens when $\Omega \in [\Omega_\text{n} - \Delta\Omega_\text{n}, \Omega_\text{n} + \Delta\Omega_\text{n}]$ where

$$\Delta\Omega_\text{n}(\Gamma_0) = \Gamma_0\frac{\Omega_\text{n}}{2J}. \tag{8}$$

Supplementary Figure 4b shows the numerical simulation the vicinity of four resonances $\Omega_\text{n} = \{J, J/2, J/3, J/4\}$ in the (Γ, Ω) plane. The $\Delta\Omega_\text{n}(\Gamma_0)$ is linearly proportional to the loss amplitude in the weak-loss limit. As n increases, both Ω_n and $\Delta\Omega_\text{n}$ decrease. We emphasize that the $\Delta\Omega_\text{n}(\Gamma_0)$ has larger values in the time-periodic coupling (supplementary Eq. 8) comparing with the time-periodic dissipation (supplementary Eq. 6).

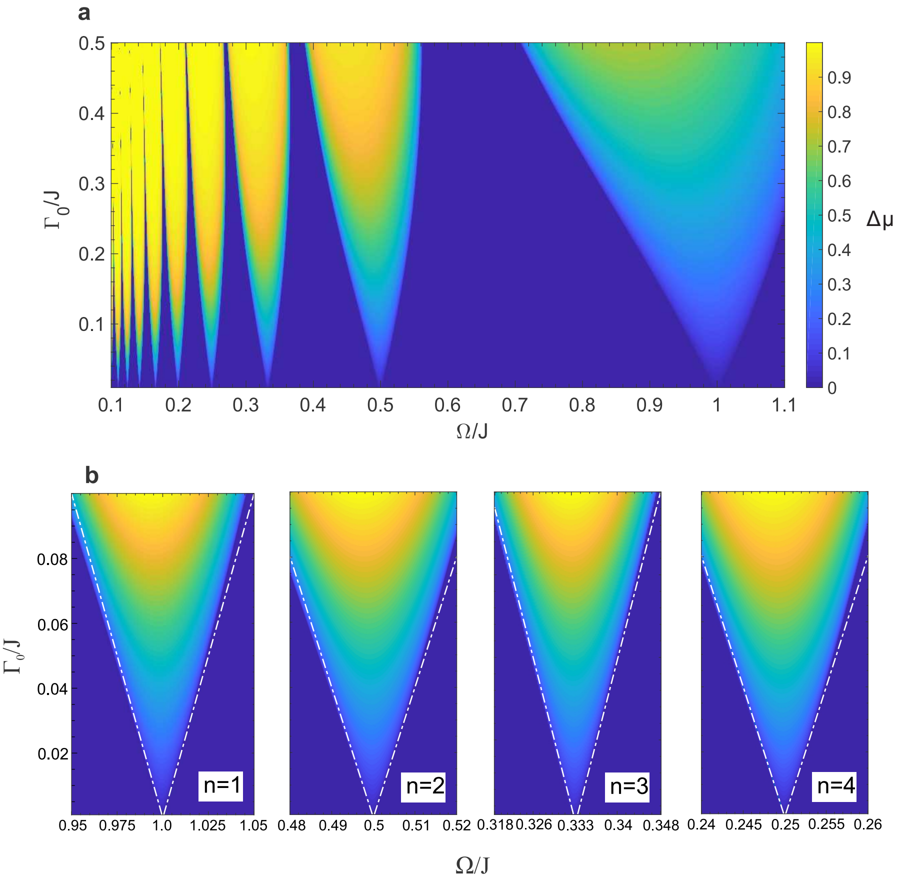

Supplementary Figure 4: $\mathcal{PT}$ phase diagram of the square-wave modulated time-periodic coupling. **a**, In the range of $\Omega/J \in \{0.1, 1.1\}$ and $\Gamma_0/J \in \{0.01, 0.5\}$. **b**, In the vicinity of first four resonances, $\Omega_\mathrm{n} = 2J/n$ for $n = \{2, 4, 6, 8\}$. The triangular region of $\mathcal{PT}$-symmetry broken phase ($\Delta\mu > 0$) separates the $\mathcal{PT}$-symmetric phase ($\Delta\mu = 0$). White lines are the analytic phase boundaries obtained from supplementary Eq.(8).

Supplementary Note 3: Effective Floquet Hamiltonian

The long-term dynamic behaviors of the system can be studied by the effective Floquet Hamiltonian H_F in a stroboscopic fashion with steps of the driving period, which is defined by

$$G(2\tau) \equiv e^{-iH_\mathrm{F}2\tau}. \tag{9}$$

Floquet Hamiltonian of time-periodic dissipation. In the time-periodic dissipation $\mathcal{PT}$ system, we derive H_F under the weak dissipation limitation, which is expressed as

$$H_\mathrm{F} = c_\mathrm{n} \begin{bmatrix} -i\gamma\cos(J\tau)\sin(\epsilon\tau) & J\cos(J\tau)\sin(\epsilon\tau) + (\epsilon\cos(\epsilon\tau) + \gamma\sin(\epsilon\tau))\sin(J\tau) \\ J\cos(J\tau)\sin(\epsilon\tau) + (\epsilon\cos(\epsilon\tau) - \gamma\sin(\epsilon\tau))\sin(J\tau) & i\gamma\cos(J\tau)\sin(\epsilon\tau) \end{bmatrix} \tag{10}$$

where c_n is a time-independent coefficients and $\epsilon = \sqrt{J^2 - \gamma^2}$. It is very obvious we can derive out the $\mathcal{PT}$ phase diagram by diagonalizing H_F. Eigenvalues

$$\lambda_\pm = c_\mathrm{n}\sqrt{\epsilon^2 - [\epsilon\cos(\epsilon\tau)\cos(J\tau) - J\sin(\epsilon\tau)\sin(J\tau)]^2} \tag{11}$$

become complex when $\sqrt{\epsilon^2 - [\epsilon\cos(\epsilon\tau)\cos(J\tau) - J\sin(\epsilon\tau)\sin(J\tau)} < 0$, which comes with the PTSB phase.

Floquet Hamiltonian of time-periodic coupling. Similar as the above, the effective Hamiltonian under the weak dissipation limitation is given by

$$H'_\mathrm{F} = c'_\mathrm{n} \begin{bmatrix} i\{\gamma - \epsilon\cot(\epsilon\tau) + e^{2\gamma\tau}[\gamma + \epsilon\cot(\epsilon\tau)]\} & -2J \\ -e^{-2\gamma\tau}2J & -i\{\gamma - \epsilon\cot(\epsilon\tau) + e^{2\gamma\tau}[\gamma + \epsilon\cot(\epsilon\tau)]\} \end{bmatrix}. \tag{12}$$

$c'_{\rm n}$ is a time-independent coefficient, and the eigenvalues of the rest matrix are given by

$$\lambda'_{\pm} = \sqrt{4e^{2+2\gamma\tau} - [\epsilon(1+e^{2\gamma\tau})\cos(\epsilon\tau) + \gamma(-1+e^{2\gamma\tau})\sin(\epsilon\tau)]^2}. \quad (13)$$

Supplementary Note 4: Mapping the $\mathcal{PT}$-symmetry phase diagram

It is relatively simple to map the phase diagram of the static dissipation, in which the PTS and PTSB phases are separated by a single exceptional point. However, the phase diagram of Floquet Hamiltonians are extraordinary rich, in which the (Γ_0, Ω) parameter-plane has an infinite number of the PTS and PTSB regions separated by lines of exceptional points [2].

We map the phase diagram by tracing the time evolution of the the normalized atom number $n'(t)$. Supplementary Figure 5a shows the time evolution of $n'(t)$ in the vicinity of the primary resonance $\Omega \approx 2J$ for a square-wave modulated dissipation. We record $n'(t)$ for five modulation frequencies. For all frequencies, the state-dependent dissipation $\Gamma_0 = 0.22J$ is an order of magnitude smaller than the transition point $2.0J$. It is found that the total atom loss-rate decreases dramatically as $\Omega \to 2J$ and reaches a minimum at the transition point. The loss rate increases again when the modulation frequency is increased further more in the PTSB phase. Supplementary Figure 5b shows $n'(\Gamma_0, \Omega)$ at a fixed time-point $t_{\rm f}$. As Γ_0 increases, the center position of the peaks of $n'(t)$ remain pegged at the transition point and the widths of $n'(t)$ increases. The peaks of $n'(t)$ indicate the appearance a long-lived mode in the $\mathcal{PT}$ Hamiltonians, and thus signal the PTSB phase. A Gaussian fit is used to extract the half-width at half-maximum (HWHM) of each peak. The inset in Supplementary Figure 5b shows that the extracted HWHM is linearly proportional to the loss strength as predicted by the theoretical model. It is confirmed that the fixed-time, frequency-dependent atom number $n'(\Omega)$ can be a good indicator to characterize the phase diagram of the Floquet Hamiltonian. However, for finite probe time, the width of the residual atom number does not equal the width of the PTSB phase predicted by theoretical calculations. The width of the residual atom number gets narrower for the longer probing times, only approaching the width of the PTSB phase for ideal infinite probe time. In our experiments, we usually extend the probe time to the point when about ten percents of the atoms left which gives us a fairly well approximation of the width of the PTSB phase.

The decrease of the decay rate of the total atom number in the PTSB phase reminds of quantum Zeno effect (QZE) previously observed in cold atom experiments [3–6]. However, the difference between our experiments and those QZE experiments exists: QZE refers to the reduction of the rate of transferring from one state to a second state by the projection measurement of the second state. Since the perturbation is the projection measurement of the second state, the reverse-transfer probability from the second state to the first state and the occupation of the second state are both treated as zero. In comparison, $\mathcal{PT}$-symmetric Hamiltonian experiments covers the crossover from the weak dissipation to the strong dissipation, and the transfer probability from the second to the first level is usually nonzero. Instead of the observation of the slow-down of the state transferring, the PTSB phase refers to the slow-down of the decay of the total atom number. In this sense, QZE can be treated as the extremely strong dissipation limit of our studies in which the strong atom loss can be treated as an irreversible projection measurement of the second level. It is also expected that QZE phenomena can expanded for more general case by using the dissipation based point of view instead of measurement based point of view, in which the analysis of $\mathcal{PT}$ phase transition can be used to study QZE-like phenomena in a pure dissipative system [7].

Supplementary Note 5: Unscaled atom number data

We present the original unscaled atom number $n'(t)$ of the experiments. Supplementary Figure 6, Supplementary Figure 7, and Supplementary Figure 8 correspond to Fig. 1,Fig. 2, and Fig. 3 of the main paper respectively.

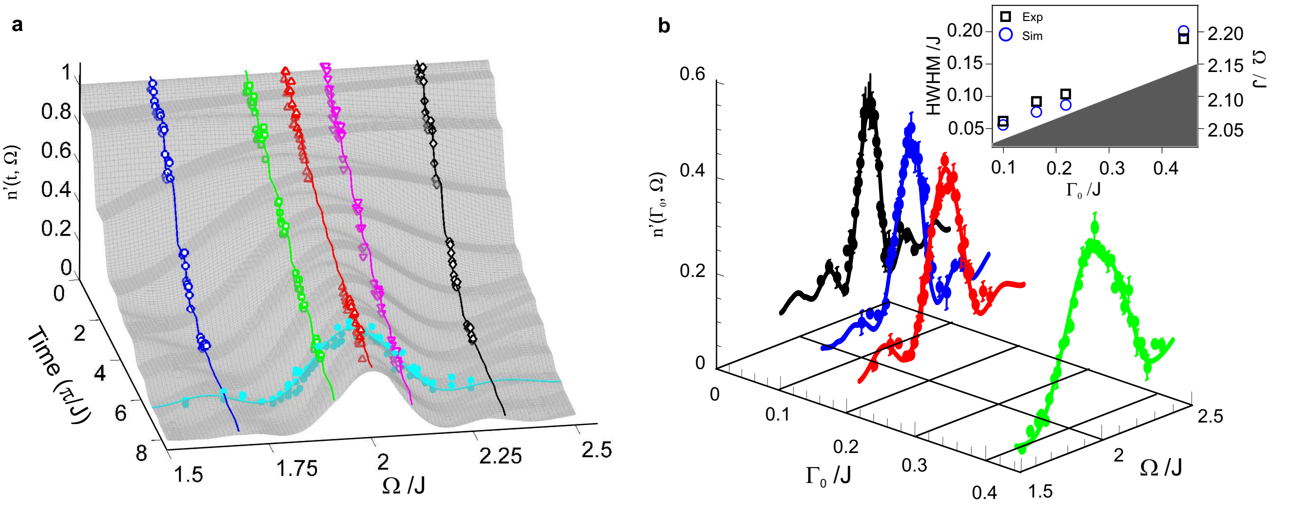

Supplementary Figure 5: Mapping the $\mathcal{PT}$-phase diagram with time-periodic dissipation. Symbols are experimental data. Gray surface and solid lines are numerical simulation with no free parameters. **a** total atom number $n'(t)$ for $\Omega = 1.67J$ (open blue circles), $\Omega = 1.91J$ (open green squares), $\Omega = 2.00J$ (open red up-triangles), $\Omega = 2.09J$ (open purple down-triangles), and $\Omega = 2.33J$ (open black diamonds). The total atom number decays most slowly at the resonance frequency, an indicator of a $\mathcal{PT}$-symmetry broken phase. A frequency-dependent measurement $n'(\Omega)$ at a fixed time-point $t_f = 7.11(\pi/J)$ (solid cyan dots) shows a sharp peak at the resonance. For all cases in **a**, $\Gamma_0 = 0.22J$. **b** $m(\Gamma_0, \Omega)$ at fixed time-points $t_m(\Gamma_0)$ shows peaks associated with the $\mathcal{PT}$-symmetry broken region: $\Gamma_0 = 0.10J$ and $t_f = 11.37(\pi/J)$ (black), $\Gamma = 0.16J$ and $t_f = 8.53(\pi/J)$ (blue), $\Gamma_0 = 0.22J$ and $t_f = 7.11(\pi/J)$ (red), and $\Gamma_0 = 0.44J$ and $t_f = 4.27(\pi/J)$ (green). The inset shows that the HWHM of the peak increases linearly with the loss amplitude Γ_0, as does the half-width $\Delta\Omega_0 = \Gamma_0/\pi$ of the $\mathcal{PT}$-symmetry broken (gray) region from the numerical simulation. The open squares are the Gaussian fits for $n'(\Omega)$ obtained from experimental data, and the open circles are the fits for numerical simulation. The error bars are the standard deviation of the measurements.

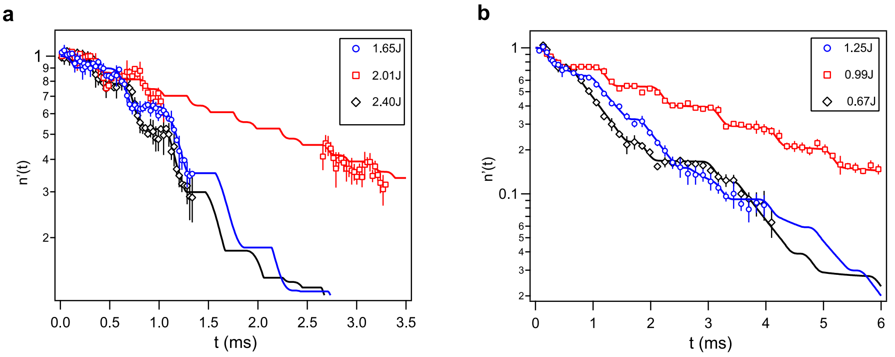

Supplementary Figure 6: Data of the unscaled atom number of Fig.1 in the main text. Symbols are experimental data, solid lines are theoretical calculation without free parameter. **a** Corresponding to Fig.1e and Fig.1f in the main text. **b** Corresponding to Fig.1h and Fig.1i in the main text. Each time point contains 4-8 individual measurements and the error bars are the standard deviation of the measurements.

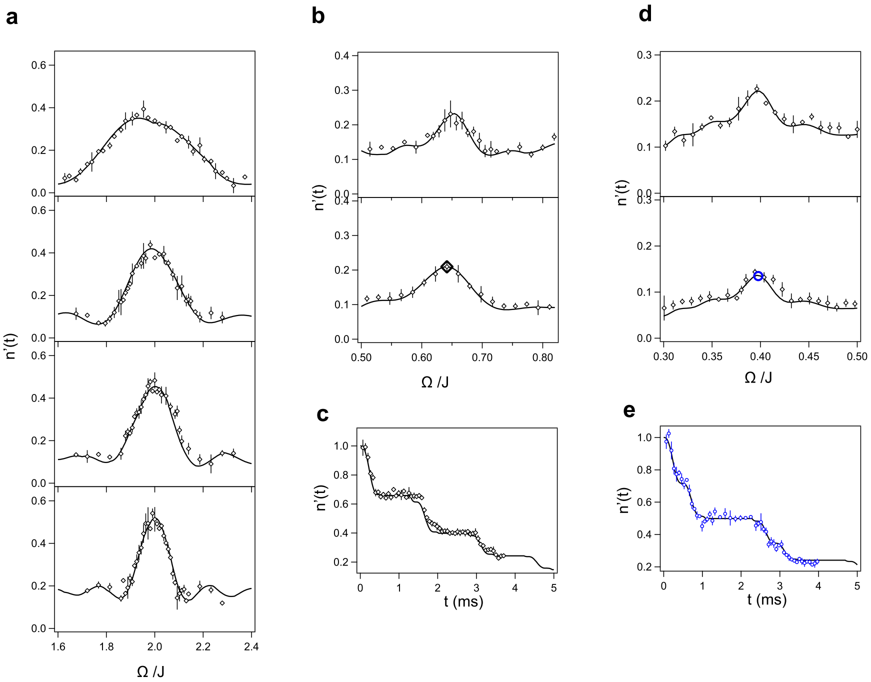

Supplementary Figure 7: Data of the unscaled atom number of Fig 2 in the main text. Symbols are experimental data, solid lines are theory with no free parameter. **a** Corresponding to Fig.2a in the main text. **b** Corresponding to Fig.2b in the main text. **c** Corresponding to Fig.2c in the main text. **d** Corresponding to Fig.2d in the main text. **e** Corresponding to Fig.2e in the main text. Each point contains 5 individual measurements and the error bars are the standard deviation of the measurements.

Supplementary References

* luole5@mail.sysu.edu.cn; yojoglek@iupui.edu

[1] T. E. Lee and Y. N. Joglekar, Phys. Rev. A **92**, 042103 (2015).
[2] Y. N. Joglekar, R. Marathe, P. Durganandini, and R. K. Pathak, Phys. Rev. A **90**, 040101 (2014).
[3] E. W. Streed, J. Mun, M. Boyd, G. K. Campbell, P. Medley, W. Ketterle, and D. E. Pritchard, Phys. Rev. Lett. **97**, 260402 (2006).
[4] M. C. Fischer, B. Gutierrez-Medina, and M. G. Raizen, Phys. Rev. Lett. **87**, 040402 (2001).
[5] B. Zhu, B. Gadway, M. Foss-Feig, J. Schachenmayer, M. L. Wall, K. R. A. Hazzard, B. Yan, S. A. Moses, J. P. Covey, D. S. Jin, et al., Phys. Rev. Lett. **112**, 070404 (2014).
[6] Y. S. Patil, S. Chakram, and M. Vengalattore, Phys. Rev. Lett. **115**, 140402 (2015).
[7] W. Kozlowski, S. F. Caballero-Benitez, and I. B. Mekhov, Phys. Rev. A **94**, 012123 (2016).

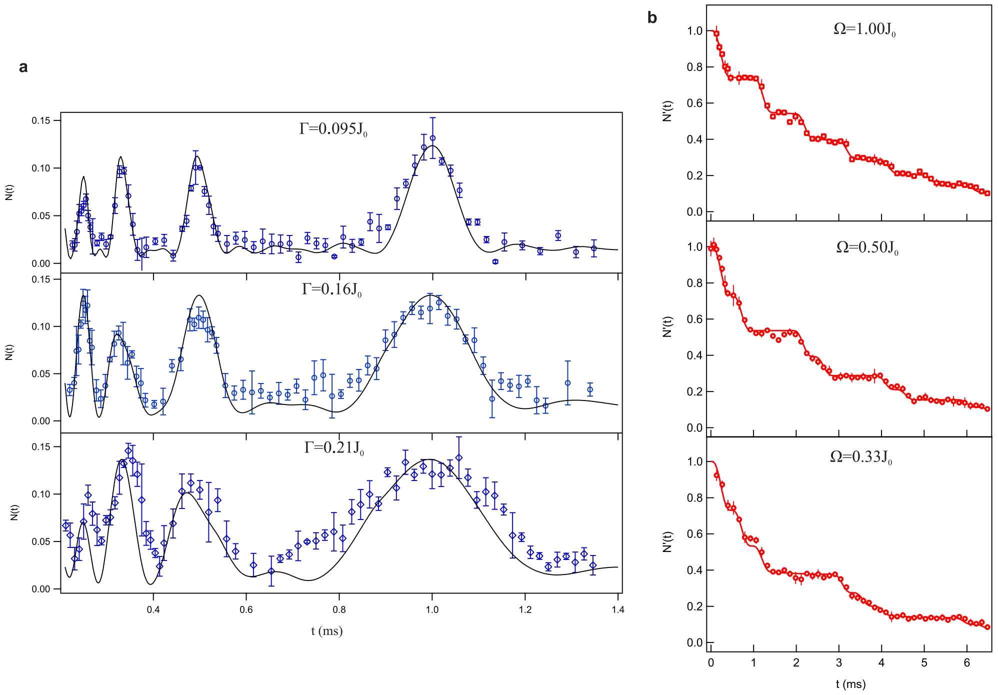

Supplementary Figure 8: Data of the unscaled atom number of Fig 3 in the main text. Symbols are experimental data, solid lines are theory with no free parameter. **a** Corresponding to Fig.3a in the main text. **b** Corresponding to Fig.3b in the main text. Each point contains 5 individual measurements and the error bars are the standard deviation of the measurements.
